# Public information sharing during public health emergencies: an adaptive-network evolutionary game

**DOI:** 10.3389/fpubh.2026.1942698

**Published:** 2026-09-08

**Authors:** Yiling Ma, Wei Feng

**Affiliations:** 1 School of Economics and Trade, Hunan University, Changsha, China; 2 School of Public Administration and Tourism, Hunan University of Technology and Business, Changsha, China

**Keywords:** adaptive network, evolutionary game, health misinformation, infodemic management, information sharing, public health emergencies, risk communication

## Abstract

**Introduction:**

Public health emergencies can generate verified sharing, opportunistic dissemination, and strategic silence within the same communication environment, yet diffusion-centered approaches offer limited explanation of how these contrasting behaviors emerge together or change as communication relationships evolve.

**Methods:**

This study develops an adaptive-network evolutionary game in which individuals choose among verified/corrective sharing, opportunistic sharing, and silence. The model links information value, verification burden, attention incentives, targeted sanctions, and participation-related liability to local interaction and adaptive rewiring.

**Results:**

Valuable and low-burden credible information favors verified sharing, whereas strong attention incentives favor opportunistic dissemination. Targeted accountability can redirect behavior toward credible participation, while generalized liability weakens good-faith sharing and, when combined with broader withdrawal incentives, can contribute to information vacuums.

**Discussion:**

By separating information quality, information absence, and structural connectivity, the study shifts attention from diffusion volume to the joint evolution of behavior and communication relationships. The findings provide a mechanism-based basis for designing public-health communication policies that constrain harmful dissemination without discouraging credible participation.

## 1 Introduction

Public health emergencies generate simultaneous demands for rapid information exchange, scientific uncertainty management, and collective protective action. Risk communication is therefore not a peripheral communication task but a core component of emergency preparedness and response. Its purpose is to enable people facing a health threat to understand uncertainty, make informed decisions, and adopt protective or preventive behaviors (1–4). The communication environment has nevertheless become increasingly difficult to govern. During disease outbreaks and other health emergencies, large volumes of accurate, uncertain, misleading, and deliberately manipulative content circulate through digital and interpersonal networks. The resulting infodemic can increase confusion, weaken trust, amplify risk-taking behavior, and undermine the public health response (1, 2).

Social media has further changed the role of the public in this process. Individuals no longer receive official messages only; they select, interpret, correct, reframe, and redistribute health information within personal networks. This distributed participation can extend the reach of timely and actionable information, especially through trusted peers and community intermediaries. It can also accelerate emotionally salient or pseudo-authoritative misinformation, while information overload, anxiety, uncertainty, and fear of responsibility may lead users to avoid information or withdraw from communication altogether (5–10). These observations point to a specific behavioral puzzle: within the same information environment during public health emergencies, some individuals engage in verified or corrective sharing, others disseminate inadequately verified content for attention or other private benefits, while still others withdraw from communication to avoid burden or risk.

Research on these processes has developed along several partly disconnected lines. Risk-communication studies explain how trust, perceived severity, uncertainty, message design, and social context influence public responses (3–6). Infodemic and health-misinformation research examines exposure, credibility judgments, corrective information sharing, media literacy, and accuracy-nudge interventions (7–14). Network and diffusion studies show that clustering, influential nodes, homophily, echo chambers, and cross-group connectivity shape the reach and persistence of information (15–22). Studies of emergency collaboration additionally emphasize the importance of interorganizational and community networks for public communication (23–25). Together, these literatures establish that information behavior is neither random nor purely individual: it is influenced by incentives, social relationships, perceived risk, institutional rules, and the evolving information environment. Recent modeling work has begun to address this dynamism from complementary angles. Research on COVID-19 information collaboration shows that communication networks change as tasks, participants, and coordination needs evolve; agent-based work demonstrates that social norms and indirect ties can redirect individual responses during public crises; and recent evolutionary-game research in public-health governance shows how changing incentives alter stakeholder strategies (26–28). These studies strengthen the case for dynamic, interaction-based explanations, but they do not jointly model verified sharing, opportunistic dissemination, silence, and endogenous adaptation of communication ties.

The unresolved problem is therefore not simply why misinformation spreads or why some individuals remain silent, but why verified/corrective sharing, opportunistic dissemination, and strategic silence can emerge and compete within the same information environment. These behaviors are not merely different levels of communication activity. They represent distinct strategic responses to information value, verification burden, private attention incentives, perceived liability, and local social interaction. Moreover, their relative attractiveness may change as neighboring behavior changes, while accumulated credibility judgments can simultaneously reshape communication ties. Explaining this coexistence and evolutionary divergence therefore requires a framework in which behavioral strategies, interaction-dependent incentives, and communication structure can evolve jointly.

Four limitations remain. First, existing diffusion-oriented studies are well suited to describing how information spreads, but they are less able to explain why individuals exposed to the same emergency environment may choose fundamentally different responses. Verified sharing, opportunistic dissemination, and silence are not simply different levels of forwarding activity; they reflect different strategic calculations about value, cost, attention, and risk. Second, many evolutionary models simplify these calculations by assigning relatively stable returns to each strategy. This makes it difficult to capture situations in which the attractiveness of a behavior changes with the composition of the surrounding network. In practice, credible sharing may become more rewarding when it is supported by credible neighbors, while opportunistic dissemination may be reinforced within like-minded clusters. The problem is therefore not only which strategy has the highest payoff, but how local interaction changes the payoff itself. Third, communication structure is often treated as a fixed background condition. This assumption overlooks the fact that credibility judgments can reshape relationships. Users may retain trusted contacts, disengage from unreliable sources, or seek new information partners. Once this occurs, behavior changes the network, and the altered network feeds back into later behavioral choices. Fourth, high communication activity does not necessarily indicate a healthy information environment. A highly connected network may still be dominated by opportunistic content, while a low-activity network may leave many individuals without access to useful information. Evaluating emergency communication therefore requires attention to information quality and local information availability, not only to diffusion volume or connectivity.

These limitations motivate the design of the present model. The first step is to treat verified/corrective sharing, opportunistic dissemination, and silence as alternative strategies rather than as different forwarding probabilities. This allows the model to examine why different forms of information behavior can emerge under the same emergency conditions. The second step is to make payoffs dependent on the strategy of the interaction partner. Credible neighbors can strengthen the return to verified sharing, opportunistic neighbors can create disruption or mutual amplification, and silence changes the relative value of active participation. As a result, the evolutionary outcome is not determined by a fixed ranking of strategies; it can change with local composition and early network conditions. The third step is to allow communication ties to adapt. Strategy updating is coupled with trust-biased rewiring, so that behavioral change affects local network structure and network change subsequently affects payoffs and exposure. This makes communication structure part of the evolutionary process rather than a fixed channel through which behavior unfolds. Finally, the model evaluates the resulting information environment using verified-information exposure, opportunistic-information exposure, information vacuum, and active connectivity. These indicators make it possible to distinguish a network that is active but dominated by low-quality information from one in which communication has largely withdrawn.

The theoretical contribution therefore lies less in combining two established modeling techniques than in changing the object of explanation. Rather than asking only how information diffuses, the model asks how different information environments emerge from the joint evolution of behavioral incentives and communication relationships. This perspective also clarifies an important asymmetry in information governance. Reducing the return to opportunistic dissemination does not automatically produce more credible participation. If regulation also raises the perceived cost or liability of good-faith sharing, the system may move toward silence instead. The model thus distinguishes between suppressing harmful dissemination and sustaining credible participation as two related but separate governance problems.

Accordingly, the central research question is: How do local incentives and changing communication relationships jointly shape the emergence and transition of verified/corrective sharing, opportunistic dissemination, and silence during public health emergencies?

## 2 Materials and methods

### 2.1 Theoretical hierarchy and conceptual framework

#### 2.1.1 From emergency risk communication to strategic information behavior

The theoretical framework is organized in three layers. At the substantive level, crisis and emergency risk communication (CERC), risk communication and community engagement (RCCE), and infodemic management define the public-health problem addressed by the model. These frameworks emphasize timely, understandable, actionable, and trustworthy communication under uncertainty, while also drawing attention to harmful information and unmet information needs (1–4, 29). They therefore establish why the availability of credible information, exposure to opportunistic content, and information absence are substantively different outcomes.

At the behavioral level, three bodies of research explain why individuals may evaluate information-sharing options differently. The social amplification of risk framework shows how interpersonal interaction can reinforce or attenuate risk interpretations, providing the basis for coordination and disruption effects (5, 6). Research on misinformation and platform incentives explains why attention, visibility, emotional salience, and peer reinforcement can increase the return to low-verification dissemination, while correction and targeted sanctions can reduce that return (7, 8, 10–14, 30–32). Information-avoidance research explains why overload, uncertainty, anticipated conflict, and responsibility can make withdrawal attractive despite the informational costs of non-participation (9).

Evolutionary game theory provides the central dynamic explanation of how these competing incentives translate into population-level behavioral change. Individuals are treated as boundedly rational actors who compare locally realized returns and probabilistically adopt strategies that perform better in their immediate environment. Adaptive-network theory extends this mechanism by allowing communication ties to change as credibility judgments accumulate (20, 21, 33–35). Strategy change therefore alters local network composition, while network adaptation changes subsequent interaction opportunities and payoffs.

The theories consequently perform different roles in the model. Public-health communication frameworks define the substantive problem and the desired information outcomes; behavioral research identifies the mechanisms entering the payoff structure; and evolutionary game and adaptive-network theory explain how those mechanisms generate dynamic regime change. The theoretical novelty of the framework lies in linking these levels rather than treating them separately: public-health communication mechanisms are translated into interaction-dependent payoffs, and those payoffs evolve together with the communication relationships through which they are generated.

#### 2.1.2 Strategy definitions and analytical boundaries

The population consists of boundedly rational individuals embedded in a communication network. At each stage, a node adopts one of three mutually exclusive strategies.

*Verified/corrective sharing (C)* includes forwarding information checked against credible sources, sharing actionable health guidance, correcting misleading claims, or adding source and uncertainty context. The defining criterion is not that every message is perfectly true, but that the actor incurs a verification effort and prioritizes public-health usefulness over private attention gain.

*Opportunistic sharing (O)* includes forwarding inadequately verified, exaggerated, emotionally amplified, or selectively framed information when attention, traffic, influence, expressive gratification, or commercial return outweigh adequate verification. It does not require deliberate fabrication. The strategy boundary is the predominance of private dissemination incentives over verification and public-health value.

*Silence (S)* includes non-participation, passive observation, delayed or withheld forwarding, and deliberate withdrawal. Silence may be individually protective when communication is burdensome or risky, but it reduces corrective capacity and may create local information deprivation (Table 1).

**Table 1:** Theoretical hierarchy and translation into the model.

Theoretical layer	Theoretical foundation	Role in the present model	Main parameters
Substantive public-health foundation	CERC and RCCE/infodemic management (1–4, 31)	Defines the value of credible communication, participation burden, and relevant public-health information outcomes	*V*: public-health value; *c*: verification cost; *l*: liability/chilling cost;
Behavioral mechanism	Social amplification of risk (5, 6)	Explains reinforcement and disruption arising from interpersonal interaction	*b*: mutual coordination benefit; *d*: disruption cost
	Misinformation and platform-incentive research (7, 8, 10–14, 28, 33, 34)	Explains incentives for low-verification dissemination and constraints created by correction and accountability	*m*: private attention gain; *f*: targeted sanction; *g*: corrective pressure; *q*: echo-chamber amplification
	Information avoidance (9)	Explains why withdrawal can become attractive and how silence changes information access	*a*: avoidance benefit; o: opportunity loss; *h*: credible-information spillover; *k*: misinformation-exposure loss
Dynamic explanatory core	Evolutionary games and adaptive networks (20, 21, 26, 27, 30)	Explains strategy adjustment and the coevolution of behavior and communication relationships	*β*: selection intensity; *ω*: adaptive rewiring rate; *η*: trust preference;

These strategies generate four propositions that guide the formal model. P1: verified/corrective sharing is self-reinforcing when credible neighbors create coordination benefits that offset verification and liability costs. P2: opportunistic sharing is self-reinforcing when attention rewards and within-cluster amplification exceed sanctions and corrective pressure. P3: silence becomes stable when avoidance returns exceed the benefits of either active strategy, particularly when credible-information spillovers are weak. P4: regulatory effects are asymmetric: targeted accountability can reduce opportunistic returns, whereas generalized liability can suppress credible participation and shift the system toward silence.

**Figure 1:**
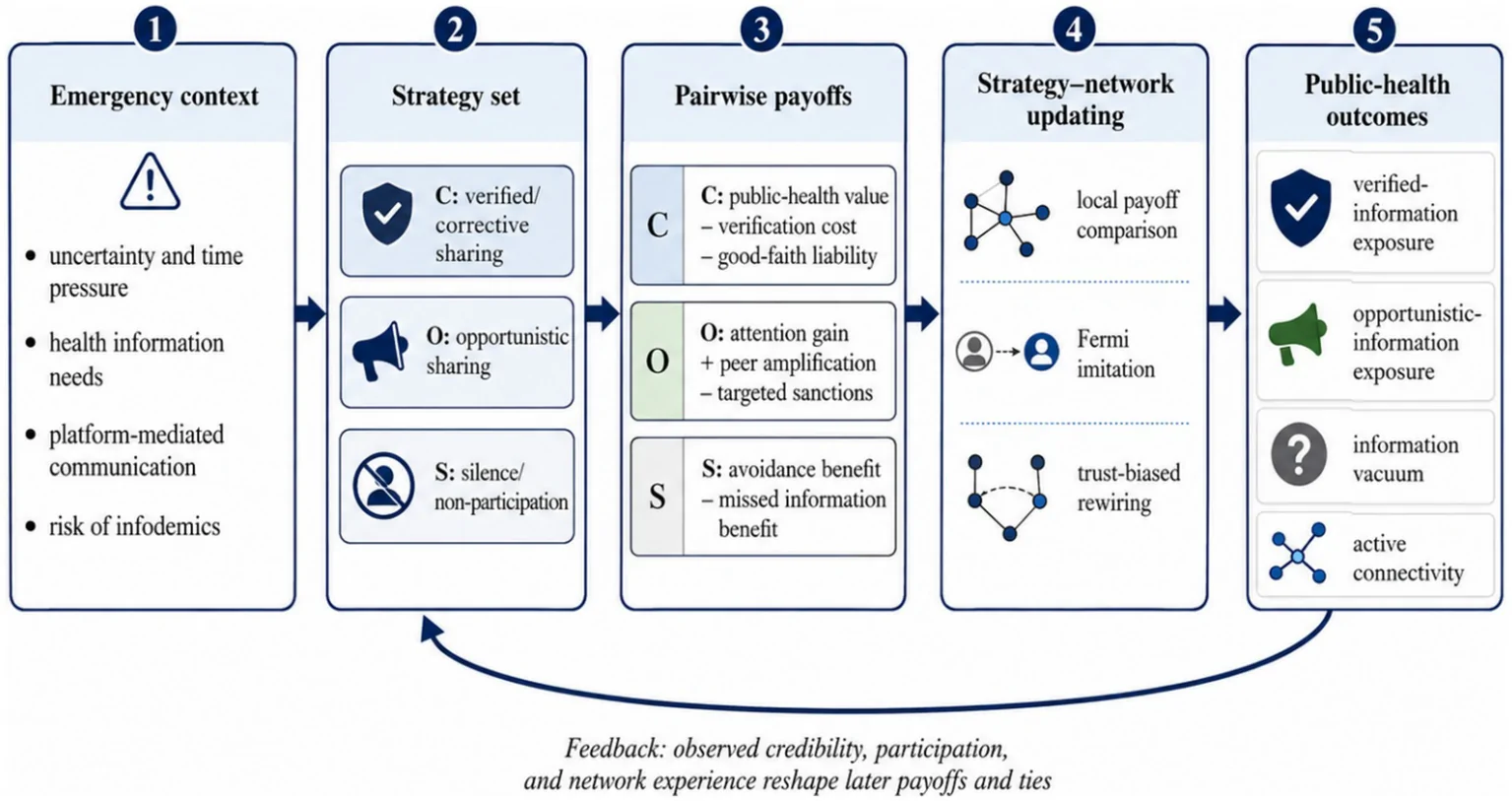
Conceptual framework of adaptive-network evolutionary dynamics in information sharing during public health emergencies Emergency conditions define the strategic environment in which individuals choose among verified/corrective sharing, opportunistic dissemination, and silence. Interaction-dependent payoffs feed into local payoff comparison, Fermi imitation, and trust-biased rewiring, producing different patterns of verified exposure, opportunistic exposure, information vacuum, and active connectivity. The feedback path indicates that accumulated credibility, participation, and network experience can reshape subsequent payoffs and relationship choices, highlighting that information outcomes arise from the joint evolution of behavior and communication structure rather than diffusion volume alone.

Figure 1 links these foundations to the model. Emergency conditions shape the payoff structure; pairwise interactions convert strategy combinations into local returns; probabilistic imitation and trust-biased rewiring update behavior and ties; and the resulting network produces distinct information-quality, deprivation, and connectivity outcomes. The feedback arrow represents accumulated experience: observed credibility, participation, and network performance reshape later payoff perceptions and relationship choices.

### 2.2 Interaction-dependent payoff structure

Let the row player be the focal individual and the column player be a neighboring individual. The payoff matrix is shown in Equation 1:


$$A=(\begin{array}{ccc} V+b-c-\ell & V-d-c-\ell & V-c-\ell \\ m-f-g & m+q-f & m-f\\ a-o+h & a-o-k & a-o \end{array}).$$ (1)

Rows and columns follow the order C , O , and S . The matrix is interaction-dependent: the return to a strategy changes with the neighbor’s strategy rather than remaining fixed. Table 2 defines the parameters.

**Table 2:** Model parameters and behavioral interpretation.

Parameter	Meaning	Behavioral interpretation
V	Public-health value of verified information	Relevance, perceived severity, usefulness, and actionability of credible information
c	Verification and cognitive cost	Time, attention, health-literacy demands, source checking, and uncertainty processing
l	Generalized liability/chilling cost	Perceived risk of blame, conflict, reputational damage, or ambiguous responsibility for good-faith sharing
b	Mutual coordination benefit	Additional return when two credible sharers reinforce consistent information and collective efficacy
d	Disruption cost	Corrective effort and conflict imposed on a credible sharer by an opportunistic neighbor
m	Private attention gain	Visibility, traffic, commercial return, emotional expression, or influence obtained from opportunistic dissemination
f	Targeted sanction	Expected platform, legal, reputational, or community penalty directed at opportunistic dissemination
g	Corrective pressure	Loss to an opportunistic sharer when a credible neighbor challenges or contextualizes the content
q	Echo-chamber amplification	Additional return when opportunistic sharers mutually reinforce one another
a	Avoidance benefit of silence	Relief from cognitive burden, conflict, responsibility, and dissemination risk
o	Opportunity loss from silence	Loss from not contributing to or benefiting from active credible communication
h	Credible-information spillover	Free-riding benefit received by a silent user connected to a credible sharer
k	Misinformation-exposure loss	Harm to a silent user exposed to opportunistic information without contributing correction

The first row captures credible participation. Its base return is V−c−ℓ ; a credible neighbor adds b , an opportunistic neighbor subtracts d , and a silent neighbor adds neither reinforcement nor disruption. The second row captures opportunistic dissemination. Its base return is m−f ; a credible neighbor adds corrective pressure −g , while an opportunistic neighbor adds amplification q . The third row captures withdrawal. Its base return is a−o ; a credible neighbor supplies spillover h , whereas an opportunistic neighbor imposes the loss k . The separation of f and ℓ is analytically essential because targeted accountability and generalized participation pressure can move the system in opposite directions.

### 2.3 Mean-field payoff derivation and evolutionary dynamics

Let *xy*, and *z* denote the proportions of *CO*, and *S*, respectively, with *x*+*y*+*z*=1. Multiplying each row of the payoff matrix by the population state vector and substituting

z=1−x−y,

gives Equation 2:


$$\begin{array}{l} \begin{array}{l} \pi_{C}=\left(V+b-c-l\right)x+\left(V-d-c-l\right)y\\ +\left(V-c-l\right)z=V-c-l+bx-dy, \end{array}\\ \pi_{O}=\left(m-f-g\right)x+\left(m+q-f\right)y+\left(m-f\right)z=m-f-gx+qy,\\ \pi_{S}=\left(a-o+\text{h}\right)x+\left(a-o-k\right)y+\left(a-o\right)z=a-o+\text{h}x-ky. \end{array}$$ (2)

These simplified expressions show the substantive role of population composition. Increasing x raises the return to credible sharing by b and lowers the return to opportunistic sharing by g , while increasing y lowers the return to credible sharing by d and raises the return to opportunistic sharing by q . Silence depends on the balance between credible spillover h and misinformation loss k .

The pairwise payoff differences are shown in Equation 3:


$$\begin{array}{l} \Delta_{CO}=\pi_{C}-\pi_{O}=\left(V-c-l-m+f\right)+\left(b+g\right)x-\left(d+q\right)y,\\ \Delta_{CS}=\pi_{C}-\pi_{S}=\left(V-c-l-a+o\right)+\left(b-\text{h}\right)x+\left(k-d\right)y,\\ \Delta_{OS}=\pi_{O}-\pi_{S}=\left(m-f-a+o\right)-\left(g+\text{h}\right)x+\left(q+k\right)y. \end{array}$$ (3)

Equation $\Delta_{\mathrm{CO}}$ makes the credibility–attention competition explicit. A larger credible cluster favors C through both coordination and correction (b+g)x , whereas a larger opportunistic cluster favors O through disruption and amplification (d+q)y . The other two differences identify when active participation can invade silence and when withdrawal becomes advantageous.

The average payoff is $\bar{\pi }=x\pi_{C}+y\pi_{O}+z\pi_{S}$ . The standard replicator system can be rewritten in pairwise-difference form as Equation 4:


$$\begin{array}{rr} \dot{x} & =x\{y(\pi_{C}-\pi_{O})+z(\pi_{C}-\pi_{S})\},\\ \dot{y} & =y\{x(\pi_{O}-\pi_{C})+z(\pi_{O}-\pi_{S})\},\\ \dot{z} & =z\{x(\pi_{S}-\pi_{C})+y(\pi_{S}-\pi_{O})\}. \end{array}$$ (4)

This form directly links changes in strategy shares to the payoff differences derived above. A strategy expands only when its weighted advantage over competing strategies is positive.

### 2.4 Equilibria and analytical implications

The equilibrium analysis is conducted on the two-dimensional simplex x + y + z = 1. At a pure-strategy vertex, local stability is determined by the two eigenvalues associated with infinitesimal invasion by the non-resident strategies. Under replicator dynamics, each invasion eigenvalue is the invading strategy’s payoff minus the resident strategy’s payoff after substituting the coordinates of that vertex. We therefore derive the three boundary equilibria directly from Equations 2, 3, as shown in Equations 5–10.

#### 2.4.1 Credible-sharing equilibrium

$$\begin{array}{rr} \pi_{C} & =V+b-c-\ell ,\\ \pi_{O} & =m-f-g,\\ \pi_{S} & =a-o+h. \end{array}$$ (5)

Substituting x = 1 and y = z = 0 into Equations 2, 3 gives the resident and mutant payoffs shown below. The two invasion eigenvalues are obtained by subtracting the resident C payoff from the O and S payoffs. Hence the credible-sharing vertex is locally asymptotically stable if and only if both invasion eigenvalues are negative:


$$\begin{array}{l} \lambda_{O|C}=(m-f-g)-(V+b-c-\ell ),\\ \lambda_{S|C}=(a-o+h)-(V+b-c-\ell ),\\ E_{C}\text{stable}\Leftrightarrow \begin{array}{c} V+b-c-\ell >m-f-g\\ V+b-c-\ell >a-o+h \end{array} \end{array}$$ (6)

#### 2.4.2 Opportunistic-sharing equilibrium

$$\begin{array}{rr} \pi_{C} & =V-c-\ell -d,\\ \pi_{O} & =m-f+q,\\ \pi_{S} & =a-o-k. \end{array}$$ (7)

Substituting y = 1 and x = z = 0 gives the three payoffs below. The relevant invasion eigenvalues compare rare C and S mutants with the resident O strategy, yielding:


$$\begin{array}{l} \lambda_{C|O}=(V-c-\ell -d)-(m-f+q),\\ \lambda_{S|O}=(a-o-k)-(m-f+q),\\ E_{C}\text{stable}\Leftrightarrow \begin{array}{c} m-f+q>V-c-\ell -d\\ m-f+q>a-o-k \end{array} \end{array}$$ (8)

#### 2.4.3 Silence equilibrium

$$\begin{array}{c} \pi_{C}=V-c-\ell ,\\ \pi_{O}=m-f,\\ \pi_{S}=a-o. \end{array}$$ (9)

Substituting z = 1 and x = y = 0 gives the stand-alone payoffs of the three strategies. The invasion eigenvalues for rare C and O mutants are therefore:


$$\begin{array}{c} \lambda_{C|S}=(V-c-\ell )-(a-o),\\ \lambda_{O|S}=(m-f)-(a-o),\\ E_{S}\text{stable}\Leftrightarrow \begin{array}{c} a-o>V-c-\ell ,\\ a-o>m-f. \end{array} \end{array}$$ (10)

These boundary calculations make the stability conditions transparent. Credible dominance requires the value and coordination return of verified/corrective sharing to exceed both opportunistic and silent alternatives; opportunistic dominance requires attention and amplification returns to withstand correction and sanctions; and silence is stable only when its avoidance return exceeds both active strategies’ stand-alone returns.

#### 2.4.4 Interior equilibrium

At an interior equilibrium all three strategy shares are strictly positive. Stationarity of the replicator system therefore requires the three strategy payoffs to be equal. Equating the credible and opportunistic payoffs and rearranging gives Equation 11:


$$\begin{array}{c} \pi_{C}=\pi_{O}\Leftrightarrow V-c-\ell +\mathrm{bx}-\mathrm{dy}=m-f-\mathrm{gx}+\mathrm{qy},\\ \Leftrightarrow (b+g)x-(d+q)y=C_{1},\\ C_{1}=m-f-V+c+\ell . \end{array}$$ (11)

Equating the credible and silence payoffs and rearranging in the same way gives Equation 12:


$$\begin{array}{c} \pi_{C}=\pi_{S}\Leftrightarrow V-c-\ell +\mathrm{bx}-\mathrm{dy}=a-o+\mathrm{hx}-\mathrm{ky},\\ \Leftrightarrow (b-h)x+(k-d)y=C_{2},\\ C_{2}=a-o-V+c+\ell . \end{array}$$ (12)

The two independent equal-payoff relations form a 2×2 linear system in the two independent equilibrium shares. Writing the system in matrix form also makes the determinant condition explicit, as shown in Equation 13:


$$\begin{array}{c} {}[\begin{array}{cc} b+g & -(d+q)\\ b-h & k-d \end{array}][\begin{array}{c} x^{*}\\ y^{*} \end{array}]=[\begin{array}{c} C_{1}\\ C_{2} \end{array}],\\ \Delta =\det\;[\begin{array}{cc} b+g & -(d+q)\\ b-h & k-d \end{array}],\\ \Delta =(b+g)(k-d)+(d+q)(b-h). \end{array}$$ (13)

If the determinant is nonzero, the coefficient matrix is nonsingular. Applying Cramer’s rule to the two columns gives the unique candidate interior solution, as shown in Equation 14:


$$\begin{array}{c} x^{*}=\frac{\det\;[\begin{array}{cc} C_{1} & -(d+q)\\ C_{2} & k-d \end{array}]}{\Delta }=\frac{C_{1}(k-d)+C_{2}(d+q)}{\Delta },\\ y^{*}=\frac{\det\;[\begin{array}{cc} b+g & C_{1}\\ b-h & C_{2} \end{array}]}{\Delta }=\frac{C_{2}(b+g)-C_{1}(b-h)}{\Delta },\\ z^{*}=1-x^{*}-y^{*}. \end{array}$$ (14)

The candidate is admissible only when all three equilibrium shares are positive. For the local-stability calculation, define two payoff-difference functions, P for credible versus opportunistic sharing and Q for credible sharing versus silence. The first two replicator equations can then be reduced as follows, as shown in Equation 15:


$$\begin{array}{c} P(x,y)=\pi_{C}-\pi_{O},\;\;\;\;Q(x,y)=\pi_{C}-\pi_{S},\\ \dot{x}=x[\mathrm{yP}+\mathrm{zQ}],\\ \dot{y}=y[-\mathrm{xP}+z(Q-P)],\;\;\;\;z=1-x-y. \end{array}$$ (15)

At the interior equilibrium both payoff-difference functions equal zero. Their partial derivatives follow directly from Equations 5, 6. Differentiating the reduced system with respect to the two independent shares and then substituting the equilibrium shares gives the Jacobian, as shown in Equation 16:


$$J^{*}=[\begin{array}{cc} x^{*}\;[y^{*}(b+g)+z^{*}(b-h)] & x^{*}\;[-y^{*}(d+q)+z^{*}(k-d)]\\ y^{*}\;[-x^{*}(b+g)-z^{*}(g+h)] & y^{*}\;[-x^{*}(d+q)+z^{*}(q+k)] \end{array}].$$ (16)

Using the simplex identity for the three equilibrium shares, the determinant and trace simplify to Equation 17:

$$\begin{array}{c} \det(J^{*})=x^{*}y^{*}z^{*}\;\Delta ,\\ \mathrm{tr}(J^{*})=x^{*}y^{*}(b+g+d+q)+x^{*}z^{*}(b-h)+y^{*}z^{*}(q+k). \end{array}$$ (17)

Because all three equilibrium shares are positive, the determinant of the Jacobian has the same sign as the coefficient determinant. The standard two-dimensional local-stability criterion is therefore a positive determinant and a negative trace. In this model, that is equivalent to a positive coefficient determinant together with a negative Jacobian trace. A negative coefficient determinant implies a saddle; a positive coefficient determinant with a positive trace implies local instability. These conditions distinguish existence from local stability and connect the analytical solution to the basin structure observed in the simulations.

### 2.5 Local strategy updating and adaptive network rewiring

The network model relaxes the well-mixed assumption. The payoff of node i is the average payoff from interactions with its current neighbors, as shown in Equation 18:

$$\Pi_{i}=\frac{1}{k_{i}}\sum_{j\in N_{i}}a_{s_{i}s_{j}},$$ (18)

where $N_{i}$ is the neighborhood, $k_{i}$ is degree, and $s_{i}$ is the node’s current strategy. Average rather than accumulated payoff is used so that high-degree nodes do not receive a mechanical payoff advantage solely because they have more neighbors.

At each elementary update, a focal node i and a randomly selected neighbor j compare payoffs. Node i adopts j ’s strategy with the Fermi probability (36–38), as shown in Equation 19:

$$P_{i\leftarrow j}=\frac{1}{1+\exp[-\beta (\Pi_{j}-\Pi_{i})]}.$$ (19)

The parameter β is selection intensity. As β→0 , updating approaches random imitation; larger values make behavior increasingly responsive to payoff differences while retaining stochastic exploration.

After strategy comparison, the sampled tie may be revised. A tie to an opportunistic neighbor receives dissatisfaction $D_{\mathrm{ij}}=1$ when the focal node is not opportunistic; a tie to a silent neighbor receives $D_{\mathrm{ij}}=0.40$ when the focal node is active; other discordant ties receive $D_{\mathrm{ij}}=0.15$ ; and same-strategy ties receive zero. The tie is rewired with probability $\omega D_{\mathrm{ij}}$ . A replacement partner is chosen from non-neighbors according to Equation 20:

$$P(i\to r)=\frac{\exp(\eta \tau_{r})}{\sum_{u\in C_{i}}\exp(\eta \tau_{u})},$$ (20)

where $C_{i}$ is the candidate set and $\tau_{C}=1$ , $\tau_{S}=0.35$ , and $\tau_{O}=0$ . This rule represents a probabilistic preference for credible and responsive ties rather than deterministic segregation. Strategy adaptation changes local composition, and rewiring changes subsequent payoffs, producing genuine strategy–network coevolution.

### 2.6 Simulation design and parameter justification

#### 2.6.1 Network and updating parameters

The main simulation uses a Watts–Strogatz small-world network because emergency communication commonly combines clustered local relationships with a smaller number of long-range links, and this topology captures both properties parsimoniously (20). The baseline network contains *N* = 250 nodes with mean degree *K* = 8. A Watts–Strogatz rewiring probability *p* = 0.10 preserves substantial local clustering while introducing shortcuts; it therefore avoids both a regular lattice and a nearly random graph. Initial strategy counts are 83, 83, and 84, producing an approximately neutral one-third allocation without preassigning dominance.

The selection intensity is β=1.5 . This intermediate value makes a one-unit payoff advantage consequential but not deterministic: the corresponding Fermi adoption probability is approximately 0.82, while equal payoffs still yield probability 0.50. The adaptive rewiring rate is ω=0.08 , so even the most dissatisfying sampled tie has at most an 8% rewiring probability per elementary update. Strategy adjustment is therefore faster than structural change, preventing the network from instantaneously sorting by strategy. Trust preference is η=1.5 ; relative to an opportunistic candidate, a credible candidate receives weight $e^{1.5}\approx 4.48$ and a silent candidate receives weight $e^{0.525}\approx 1.69$ . This creates a meaningful but non-exclusive preference for credibility.

Each run lasts 100 Monte Carlo steps, with one step equal to N elementary updates. In the generated trajectories, the decisive scenarios reach stable plateaus well before the end of this window, while the baseline has sufficient time to reveal stochastic path dependence. Thirty independent runs are used for the main results to estimate between-run variation. Robustness tests use 20 independent runs per topology–rewiring combination because they span 12 additional conditions.

#### 2.6.2 Parameter grounding, normalization, and baseline selection

The payoff parameters are normalized representations of behavioral mechanisms rather than empirical coefficients estimated from a specific emergency event. Previous empirical studies are used to determine the direction and substantive meaning of the parameters, but not to assign exact numerical effect sizes. Research on emergency risk communication, message design, and accuracy interventions supports the assumption that useful and credible information increases the attractiveness of verified sharing, whereas verification effort and perceived responsibility increase its cost. Studies of social amplification and networked information behavior support reinforcement among similar information behaviors and disruption between competing forms of communication. Research on health-information avoidance provides the basis for treating cognitive burden, uncertainty, conflict, and perceived responsibility as incentives for withdrawal. These studies therefore constrain the signs and relative directions of the payoff components.

Exact numerical calibration is more difficult because the relevant empirical literature reports heterogeneous quantities—such as behavioral probabilities, experimental treatment effects, survey associations, and network statistics—that do not share a common utility scale. Converting these estimates directly into comparable payoff values would introduce a degree of precision that the available evidence does not support. We therefore use a normalized scale and treat the parameters as relative behavioral incentives. The public-health value of verified information is set to *V* = 1.00 in the baseline and serves as the reference magnitude against which the remaining payoff components are scaled. Table 3 reports the baseline value of each payoff parameter together with the range of values actually used across the policy scenarios, thereby making the numerical scope of the model explicit.

**Table 3:** Baseline values and scenario ranges of payoff parameters.

Parameter	Baseline	Values used across scenarios	Basis for variation
V	1.00	1.00–1.35	Information usefulness and actionability
c	0.45	0.25–0.45	Verification and cognitive burden
ℓ	0.15	0.08–0.65	Good-faith liability/chilling pressure
b	0.35	0.35	Credible coordination reinforcement
d	0.35	0.35	Disruption from opportunistic neighbors
m	0.75	0.65–1.15	Private attention return
f	0.35	0.25–0.75	Targeted accountability
g	0.30	0.30–0.45	Corrective pressure
q	0.25	0.25–0.45	Opportunistic peer amplification
a	0.55	0.45–0.85	Avoidance benefit
o	0.35	0.20–0.45	Opportunity loss from silence
h	0.20	0.20	Credible-information spillover
k	0.25	0.25	Opportunistic-information exposure loss

Baseline values are then chosen subject to the analytical conditions derived in section 2.4. The purpose of the baseline is not to reproduce a particular historical event, but to represent a plausible competitive environment in which verified and opportunistic sharing are both locally viable while silence is initially less attractive. At equal initial shares, credible and opportunistic sharing are deliberately close in expected payoff, placing the system near the analytical boundary and allowing local composition and stochastic updating to influence which basin is reached.

Counterfactual scenarios are then constructed by changing only the parameters associated with the mechanism of interest while holding all remaining parameters at their baseline values. Table 4 shows how these parameter changes are combined into six policy and incentive scenarios and clarifies the behavioral rationale and analytical regime targeted by each scenario. Supportive communication increases public value and lowers verification and good-faith liability burdens; targeted accountability raises the specific cost of opportunistic dissemination without increasing the cost of credible sharing; blanket regulation combines stronger sanctions with higher generalized liability and avoidance incentives; the attention-economy scenario increases private attention returns and peer amplification while weakening constraint; withdrawal pressure makes non-participation more attractive; and the integrated intervention combines changes across the principal mechanisms. The integrated-intervention scenario represents a policy package in which several mechanisms are adjusted simultaneously. Its results therefore indicate the aggregate outcome of the combined intervention. Because the model does not vary these components independently within this scenario, the marginal contribution of any single policy measure cannot be identified.

**Table 4:** Scenario design and parameter-selection rationale.

Scenario	Parameters changed from baseline	Rationale for change	Analytical regime targeted
Baseline	None	Near-competitive C – O payoffs and initially unattractive silence permit path dependence	Bistability of $E_{C}$ and $E_{O}$
Supportive communication	V=1.35 , c=0.30 , ℓ=0.10	More useful and shareable official information, lower checking burden, and safer good-faith participation	$E_{C}$
Targeted accountability	f=0.65 , g=0.45	Stronger sanction and correction directed specifically at opportunistic dissemination	$E_{C}$
Blanket regulation	f=0.75 , ℓ=0.65 , a=0.65 , o=0.25	Broad responsibility pressure makes both active strategies costly and increases withdrawal incentives	$E_{S}$
Attention economy	m=1.15 , q=0.45 , f=0.25	High visibility rewards, stronger peer amplification, and weak constraint on opportunistic content	$E_{O}$
Withdrawal pressure	a=0.85 , o=0.20 , ℓ=0.35	High relief from non-participation, low cost of silence, and elevated participation burden	Predominantly $E_{S}$
Integrated intervention	V=1.25 , c=0.25 , ℓ=0.08 , f=0.65 , g=0.45 , m=0.65 , a=0.45 , o=0.45	Simultaneously improves credible sharing and weakens both opportunistic and silent alternatives	$E_{C}$

#### 2.6.3 Numerical experiments, sensitivity analysis, and validation

Mean-field regime maps are generated from equal initial shares on two 91 × 91 parameter grids. The value–attention map varies V from 0.55 to 1.55 and m from 0.35 to 1.35, while the regulation-design map varies ℓ and f from 0 to 0.90. Each grid is integrated for 1,800 Euler steps at Δ*t* = 0.06, corresponding to 108 normalized time units, and each cell is classified by the strategy with the largest final share.

A local sensitivity analysis was conducted for four payoff parameters that are central to the main conclusions: public-health value V, private attention gain m, targeted sanction f, and generalized liability/chilling cost ℓ. Each parameter was varied independently at 80, 90, 100, 110, and 120% of its baseline value, while all other payoff and network parameters were held constant. The resulting ranges were *V* = 0.80–1.20, *m* = 0.60–0.90, *f* = 0.28–0.42, and ℓ = 0.12–0.18. These bounds are symmetric local perturbations rather than empirical confidence intervals. For each parameter level, 20 independent Watts–Strogatz network simulations were performed using *N* = 250, *K* = 8, *p* = 0.10, *β* = 1.5, ω = 0.08, η = 1.5, and 100 Monte Carlo steps. The same replicate seed schedule was used across parameter levels, and final strategy proportions together with the four network-level information outcomes were recorded.

Structural robustness is examined separately by replacing the Watts–Strogatz network with Erdős–Rényi random and Barabási–Albert scale-free networks and by varying the adaptive rewiring rate ω across 0, 0.04, 0.08, and 0.16. These tests retain *N* = 250, *K* = 8, *β* = 1.5, η = 1.5, the 100-step horizon, and 20 independent runs per topology–rewiring condition. This design distinguishes uncertainty in behavioral payoffs from uncertainty in network structure and adaptation speed.

Population-size scaling was evaluated separately to test whether the network results are an artifact of the baseline N. We repeated the Watts–Strogatz simulations at *N* = 125, 250, and 500, corresponding to one-half, the baseline, and twice the main network size. Mean degree *K* = 8, network rewiring probability *p* = 0.10, selection intensity *β* = 1.5, adaptive rewiring rate ω = 0.08, trust preference η = 1.5, and the 100-step horizon were held fixed, thereby preserving the local interaction scale while changing only population size. Four representative regimes—Baseline, Attention economy, Blanket regulation, and Integrated intervention—were evaluated with 20 independent runs at each N using the same replicate seed schedule across sizes. Final strategy proportions and the four public-health network outcomes were compared.

### 2.7 Outcome measures and measurement rationale

Final strategy proportions identify which behavior dominates, but they do not show whether information is available locally, whether users are exposed to harmful content, or whether active communication remains structurally connected. Four complementary measures are therefore used. They correspond to four distinct dimensions of public-health information performance: credible availability, harmful exposure, information deprivation, and structural reach.

Let $G=(V_{G},E)$ be the communication network, $N_{i}$ the neighbors of node i , and I() an indicator function. Verified-information exposure is

$$\mathrm{VE}=\frac{1}{N}\sum_{i=1}^{N}I(s_{i}=C\;\text{or}\exists j\in N_{i}:s_{j}=C).$$ (21)

This measure is selected because a user can access credible information either by sharing it directly or through at least one immediate credible source. It captures local availability rather than assuming that every message reaches the entire network.

Opportunistic-information exposure is

$$\mathrm{OE}=\frac{1}{N}\sum_{i=1}^{N}I(s_{i}=O\;\text{or}\exists j\in N_{i}:s_{j}=O).$$ (22)

It measures contamination risk: a network may be highly active and connected while exposing nearly everyone to inadequately verified content. Reporting OE separately prevents activity from being mistaken for information quality.

Information vacuum is

$$\mathrm{IV}=\frac{1}{N}\sum_{i=1}^{N}I(s_{j}=S\;\text{for}\;\mathrm{all}\;j\in N_{i}\cup \{i\}).$$ (23)

This measure identifies nodes located in closed neighborhoods with no active source. It operationalizes unmet information need and local communication deprivation, which are central concerns in risk communication and infodemic management.

Finally, let $G_{A}$ be the subgraph induced by active nodes with $s_{i}\in \{C,O\}$ . The active giant component is

$$\mathrm{AGC}=\frac{|\mathrm{LCC}(G_{A})|}{N},$$ (24)

where LCC is the largest connected component. AGC measures whether active communication can form a system-level pathway. It is interpreted jointly with VE and OE , because structural reach alone cannot distinguish a credible network from an opportunistic one.

Together, the four measures form a nonredundant diagnostic set: VE evaluates desirable access, OE evaluates harmful access, IV evaluates absence, and AGC evaluates reach. Means and normal-approximation 95% Monte Carlo confidence intervals are reported across independent runs. Complete code, parameter files, and generated datasets are included in Supplementary material.

## 3 Results

### 3.1 Analytical regimes and evolutionary implications

The payoff derivation clarifies how each regime is generated. In $\Delta_{\mathrm{CO}}$ , credible sharing gains two advantages as the credible share x rises: mutual coordination contributes bx , and corrective pressure reduces the relative attractiveness of opportunistic dissemination by gx . Their combined effect is (b+g)x . By contrast, a larger opportunistic share y simultaneously imposes disruption on credible sharers and creates echo-chamber reinforcement for opportunistic sharers, producing the opposing term −(d+q)y . The boundary between the two active strategies is therefore endogenous to population composition rather than determined by a fixed payoff ranking.

The stability conditions identify distinct policy levers. $E_{C}$ becomes easier to stabilize when V , b , f , or g increases and when c , l , m , a , or h decreases relative to the competing terms. $E_{O}$ is promoted by m and q , but weakened by f and by credible corrective pressure. $E_{S}$ is promoted by high avoidance benefit a , low opportunity loss o , and low stand-alone returns to both active strategies. Importantly, f and l enter different inequalities: increasing f directly reduces opportunistic returns, whereas increasing l reduces credible returns. This is why targeted accountability can support credible sharing while generalized liability can produce silence.

The baseline satisfies the local stability conditions for both $E_{C}$ and $E_{O}$ , but not $E_{S}$ . It is therefore not a weakly specified middle case; it is an intentionally bistable case. At equal shares, credible sharing has only a 0.017 payoff advantage over opportunistic sharing. Small early differences in local clustering or stochastic imitation can consequently be amplified, leading different runs toward different active equilibria. This analytical result anticipates the wide between-run interval observed in the network simulation.

**Figure 2:**
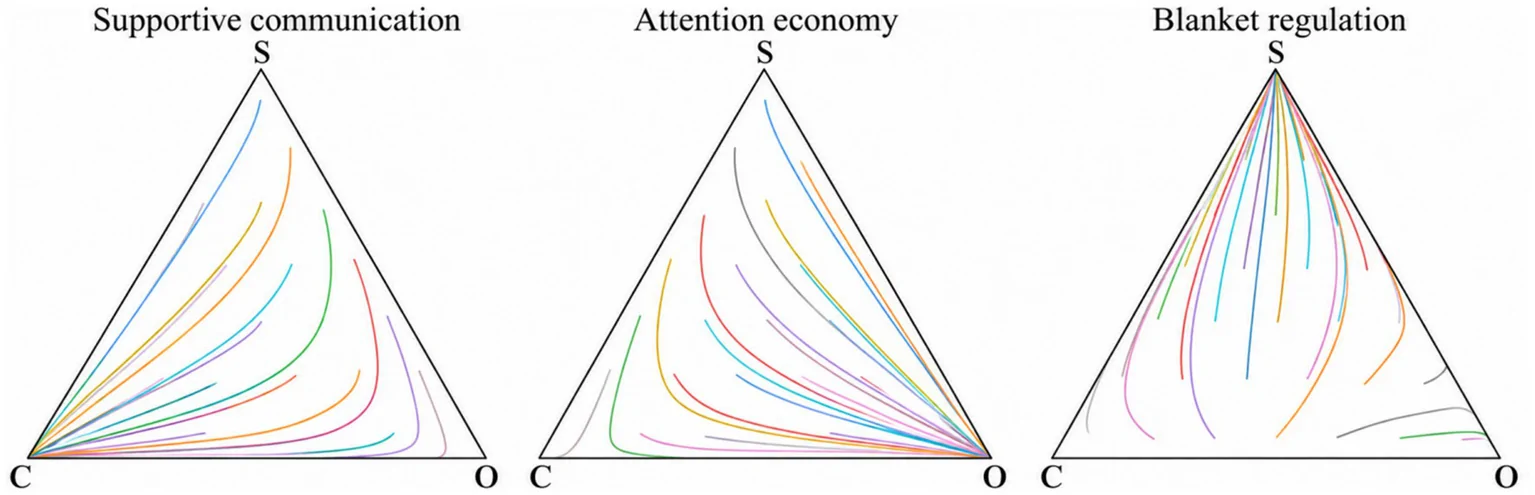
Mean-field simplex trajectories under supportive communication, attention-economy, and blanket-regulation scenarios. The trajectories show three qualitatively different basins: supportive communication moves the system toward verified/corrective sharing, attention incentives favor opportunistic dissemination, and blanket regulation shifts the system toward silence. The comparison illustrates why the effect of regulation depends on which behavioral costs and incentives are changed rather than on regulatory intensity alone.

Figure 2 displays mean-field trajectories from multiple initial states. Under supportive communication, all shown trajectories bend toward the C vertex because the increase in V and reductions in c and l make credible sharing resistant to both opportunistic and silent invasion. Under the attention economy, trajectories move toward O because private gain and within-group reinforcement dominate correction and sanction. Under blanket regulation, the initial decline of opportunistic sharing does not produce credible coordination; instead, generalized liability and avoidance make both active strategies less attractive, and trajectories converge to S . The figure therefore illustrates three different basins rather than a single monotonic response to “more regulation.”

### 3.2 Strategy trajectories across scenarios

**Figure 3:**
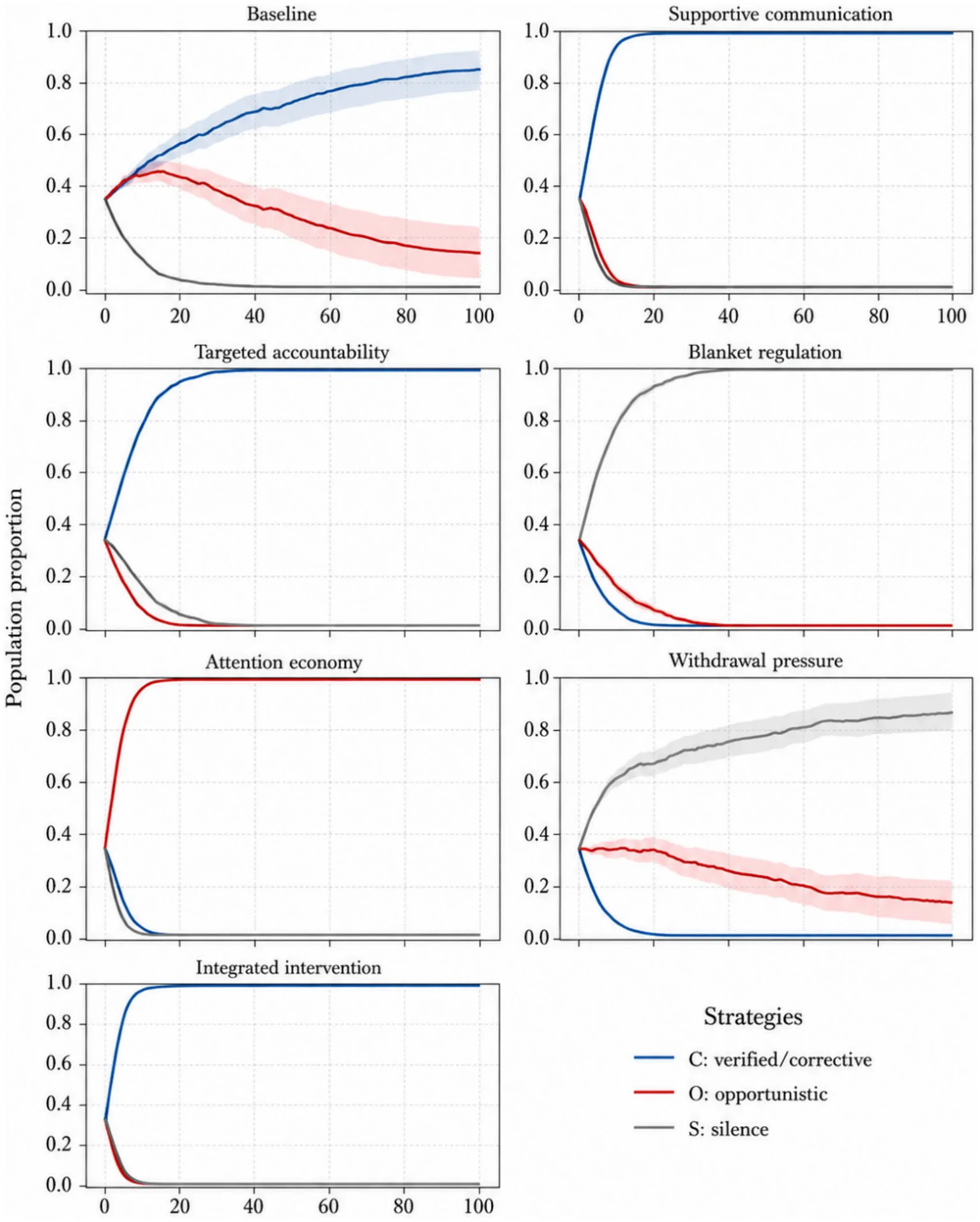
Mean strategy trajectories across 30 independent network simulations. Lines show mean strategy proportions and shaded bands show 95% Monte Carlo confidence intervals. The widening baseline band reflects path dependence near the credible–opportunistic boundary, whereas the intervention scenarios produce more clearly separated trajectories. The figure therefore shows how local clustering and stochastic early advantages affect the realized network outcome.

Figure 3 shows the average trajectories and 95% Monte Carlo confidence intervals across 30 independent runs. The early stage is decisive. During approximately the first 10–20 steps, locally successful strategies expand through imitation and reconfigure the composition of neighboring payoffs. Once one strategy occupies most local clusters, the interaction terms reinforce its advantage and convergence accelerates.

In the baseline, silence falls rapidly from 0.336 because its initial payoff (0.183) is substantially below those of both active strategies. Credible and opportunistic sharing then compete for the vacated nodes. The mean credible share rises steadily, but the confidence band widens rather than narrows during the middle stage. This pattern is consistent with bistability: most runs converge to credible dominance, whereas a smaller set is captured by opportunistic clusters. At step 100, the final credible share is 0.881 (95% CI: 0.771–0.991), the opportunistic share is 0.119 (95% CI: 0.009–0.229), and silence is eliminated.

Supportive communication produces a rapid direct transition to credible sharing. Raising V while reducing c and l makes credible sharing advantageous against all neighbor types, so local credible clusters expand immediately and reach 90% after a mean of 7.8 steps (SD 1.0). Targeted accountability reaches the same final state but more slowly: credible sharing exceeds 90% after 15.5 steps (SD 3.9). This difference is substantively meaningful. Supportive communication improves the payoff of credible participation directly, whereas targeted accountability must first erode the local reinforcement of opportunistic clusters. The integrated intervention is fastest, reaching 90% credible sharing after 5.7 steps (SD 0.7), because it simultaneously raises credible returns and lowers both competing returns.

The attention economy reverses the direction of selection. Opportunistic sharing reaches 90% after 7.4 steps (SD 1.1) and converges to 1.000 in every run. Higher m produces a strong individual incentive to disseminate attention-generating content, and higher q turns opportunistic neighborhoods into reinforcing clusters. Blanket regulation produces complete silence after 16.6 steps (SD 4.8). Opportunistic content initially declines because f is high, but credible sharing cannot replace it because l is also high and the net avoidance return is favorable. Withdrawal pressure produces a less uniform version of the same mechanism. At step 100, silence averages 0.881 (95% CI: 0.784–0.979), while opportunistic sharing averages 0.119 (95% CI: 0.021–0.216). Residual opportunistic clusters survive in some runs because withdrawal removes nearby corrective competition without eliminating the private attention return. Table 5 summarizes the final strategy proportions across all seven scenarios.

**Table 5:** Final strategy proportions across 30 independent runs.

Scenario	Verified/corrective sharing, C	Opportunistic sharing, O	Silence, S
Baseline	0.881 (0.771–0.991)	0.119 (0.009–0.229)	0.000
Supportive communication	1.000	0.000	0.000
Targeted accountability	1.000	0.000	0.000
Blanket regulation	0.000	0.000	1.000
Attention economy	0.000	1.000	0.000
Withdrawal pressure	0.000	0.119 (0.021–0.216)	0.881 (0.784–0.979)
Integrated intervention	1.000	0.000	0.000

### 3.3 Parameter sensitivity and joint regime boundaries

**Figure 4:**
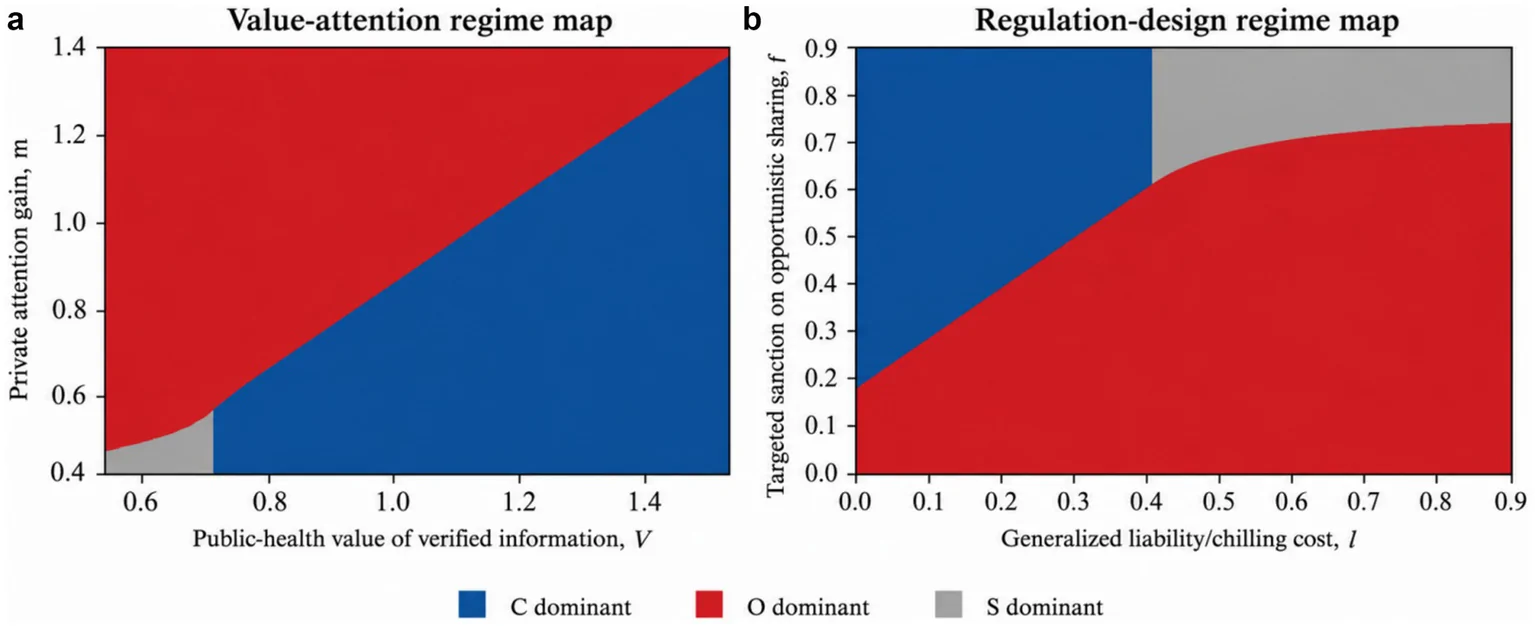
Two-dimensional parameter sensitivity and mean-field regime boundaries. **(a)** Varies public-health information value V and private attention gain m; **(b)** varies generalized liability ℓ and targeted sanction f, with all remaining parameters held at baseline. Each cell is classified by the largest final strategy share. The maps show that higher information value can offset stronger attention incentives, while targeted accountability supports credible sharing most reliably when generalized participation costs remain limited.

The value–attention map in Figure 4a varies V and m while holding the remaining parameters at baseline. The diagonal boundary reflects the direct competition between the public value of credible sharing and the private return to opportunistic dissemination. At low m , a moderate increase in V is sufficient to move the system into the credible regime. As m rises, increasingly large public value is required because opportunistic sharing gains both a higher base return and local amplification. The boundary is not perfectly vertical or horizontal because the final state also depends on the interaction terms b , d , g , and q .

A narrow silence region appears when both active strategies have low net returns. This result is important because low credibility does not necessarily imply high misinformation. Under some parameter combinations, the dominant failure is insufficient participation rather than active opportunism. Interventions must therefore diagnose whether the system is attention-driven or withdrawal-driven before selecting a policy lever.

Figure 4b varies targeted sanction f and generalized liability l . When l is low, increasing f moves the system from opportunistic to credible dominance because it changes the relative payoff of the two active strategies without burdening credible participation. When l becomes high, the credible region contracts even if f is also high. The upper portion of the map becomes silent because both active strategies are unattractive relative to avoidance. The map thus identifies a regulatory corridor: targeted accountability is most effective when accompanied by clear protection for good-faith sharing.

The local network sensitivity analysis gives the same directional picture around the deliberately competitive baseline. When V increases from 0.80 to 1.20, the mean final verified/corrective share rises from 0.000 at 0.8 V₀ and 0.091 at 0.9 V₀ to 0.801 at V₀, reaching 1.000 at both 1.1 V₀ and 1.2 V₀. Changing the private attention gain produces the opposite response: C equals 1.000 at 0.8 m₀ and 0.9 m₀, falls to 0.801 at m₀ and 0.023 at 1.1 m₀, and reaches 0.000 at 1.2 m₀.

Targeted sanctions also move the system in the expected direction: increasing f from 0.8f₀ to 1.2f₀ raises the mean final C share from 0.099 to 1.000. Generalized liability behaves differently. Increasing ℓ from 0.8ℓ₀ to 1.2ℓ₀ reduces mean final C from 1.000 to 0.509, with the displaced share moving primarily toward opportunistic dissemination within this local range. Thus, generalized liability directly weakens credible participation, but liability alone does not generate the silence regime under a ± 20% perturbation. Silence emerges when liability is combined with a broader payoff configuration that also lowers the attractiveness of active participation or raises the return to withdrawal. The baseline is therefore not parameter-insensitive, as expected from its placement near a regime boundary; what remains stable is the direction in which the four focal mechanisms move the system. Local sensitivity of final verified/corrective sharing to key payoff parameters are shown in Table 6.

**Table 6:** Local sensitivity of final verified/corrective sharing to key payoff parameters.

Parameter	80%	90%	100%	110%	120%
V (baseline 1.00)	0.000	0.091	0.801	1.000	1.000
m (baseline 0.75)	1.000	1.000	0.801	0.023	0.000
f (baseline 0.35)	0.099	0.397	0.801	0.899	1.000
ℓ (baseline 0.15)	1.000	0.851	0.801	0.722	0.509

### 3.4 Public-health information outcomes

**Figure 5:**
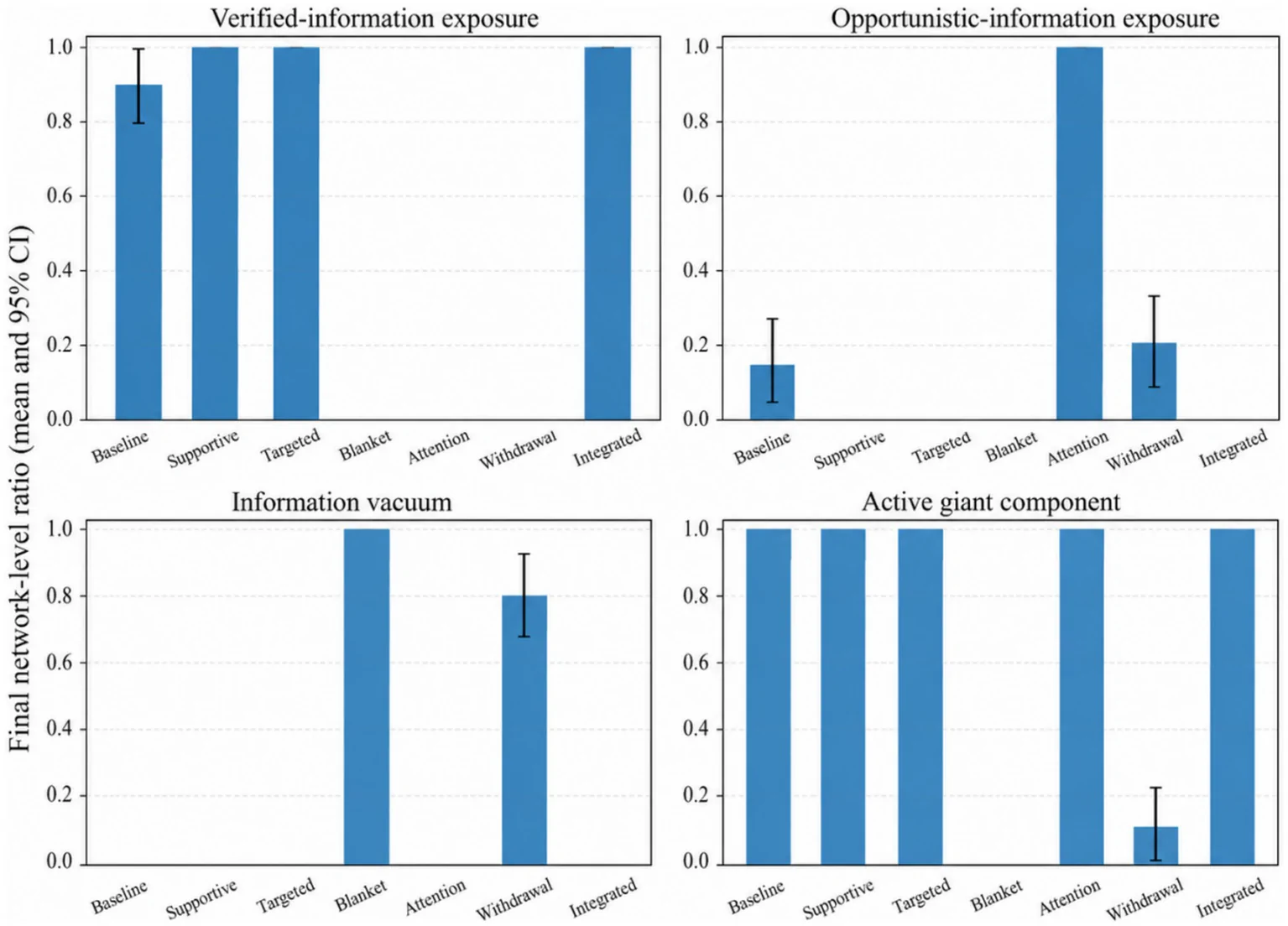
Final public-health information and connectivity outcomes across scenarios. The comparison separates verified-information exposure, opportunistic-information exposure, information vacuum, and active connectivity. It shows why connectivity alone is not a sufficient indicator of communication quality: an attention-driven network can remain fully connected while becoming entirely opportunistic, whereas withdrawal can reduce harmful exposure at the cost of extensive information deprivation.

The four outcome measures reveal differences that final strategy shares alone cannot fully express (Figure 5 and Table 7). In the baseline, verified exposure is 0.899 (95% CI: 0.790–1.000), exceeding the final credible share because some opportunistic nodes remain adjacent to credible sources. Opportunistic exposure is 0.150 (95% CI: 0.028–0.273), which is also higher than the final opportunistic share because a relatively small opportunistic cluster can expose adjacent credible nodes. The active giant component remains 1.000 and the information vacuum is zero. The baseline is therefore structurally connected and mostly credible, but it retains a nontrivial risk of local opportunistic exposure across runs.

**Table 7:** Public-health information and connectivity outcomes.

Scenario	Verified exposure	Opportunistic exposure	Information vacuum	Active giant component
Baseline	0.899 (0.790–1.000)	0.150 (0.028–0.273)	0.000	1.000
Supportive communication	1.000	0.000	0.000	1.000
Targeted accountability	1.000	0.000	0.000	1.000
Blanket regulation	0.000	0.000	1.000	0.000
Attention economy	0.000	1.000	0.000	1.000
Withdrawal pressure	0.000	0.204 (0.085–0.323)	0.796 (0.677–0.915)	0.114 (0.016–0.212)
Integrated intervention	1.000	0.000	0.000	1.000

Values are means with normal-approximation 95% Monte Carlo confidence intervals in parentheses. Zero-width intervals are omitted.

Supportive communication, targeted accountability, and the integrated intervention all produce the same final outcome profile: complete verified exposure, zero opportunistic exposure, zero information vacuum, and a fully connected active component. Their identical endpoints conceal different transition speeds, which is why trajectory measures and final outcomes should be considered jointly. Supportive communication and the integrated intervention reach the desirable state substantially faster than targeted accountability alone.

The attention-economy scenario demonstrates the limitation of connectivity as a stand-alone performance indicator. Opportunistic exposure and the active giant component both equal 1.000, while verified exposure equals zero. Every node belongs to a system-spanning active network, but that network is entirely dominated by inadequately verified, privately rewarded dissemination. A metric based only on activity, density, or reach would classify this environment as strong even though its information quality is poor.

Blanket regulation produces the opposite failure. The information vacuum equals 1.000 and the active giant component equals zero. Potential social ties still exist in the underlying network, but no node participates actively, so the communication system has no functional pathway. Under withdrawal pressure, the vacuum is incomplete but extensive: information vacuum averages 0.796 (95% CI: 0.677–0.915), opportunistic exposure averages 0.204 (95% CI: 0.085–0.323), and the active giant component falls to 0.114 (95% CI: 0.016–0.212). This combination represents fragmented islands of opportunistic activity surrounded by silent neighborhoods.

### 3.5 Strategy–network coevolution

**Figure 6:**
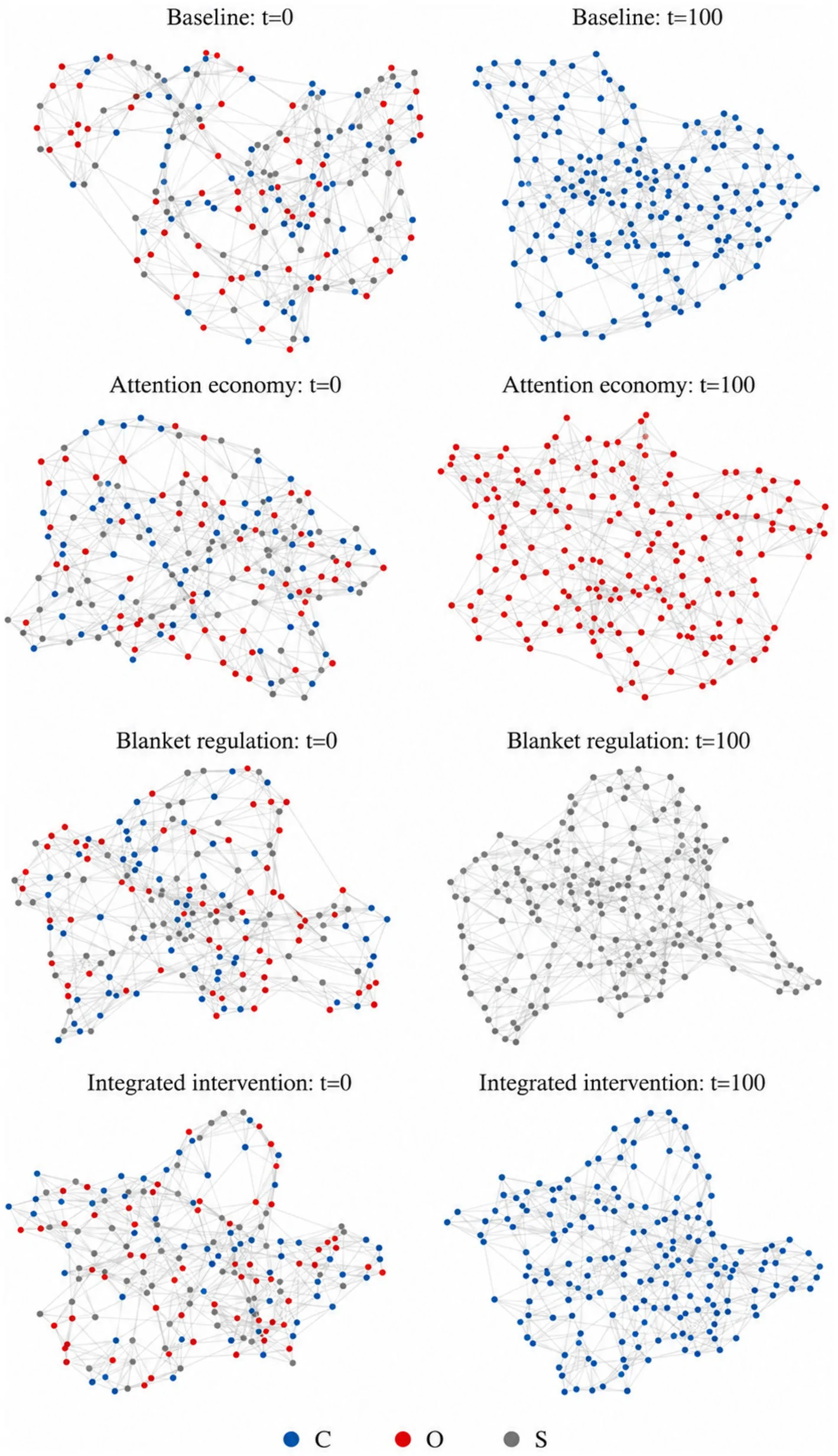
Representative network snapshots showing strategy–network coevolution. Node colors indicate information-sharing strategies and edges represent potential communication ties. The snapshots illustrate how imitation changes local strategy composition while trust-biased rewiring reorganizes ties, allowing credible or opportunistic clusters to consolidate; under blanket regulation, withdrawal occurs before an active credible backbone can form.

The network snapshots in Figure 6 show how strategy change and tie adaptation jointly reorganize the communication environment. At t=0 , all scenarios begin from the same heterogeneous strategy distribution. During the early stage, imitation changes the strategy composition of local clusters, while dissatisfaction-based rewiring selectively removes some ties to opportunistic or inactive neighbors. The new ties are biased toward credible candidates but remain probabilistic, so the process does not impose a fully segregated network.

In the baseline, credible clusters expand around locally successful nodes and gradually absorb most of the network. A small number of opportunistic clusters can nevertheless persist because the baseline permits $E_{O}$ to be locally stable. Under supportive communication and the integrated intervention, credible nodes become attractive both behaviorally and relationally: they have higher payoffs and receive greater rewiring weight. This creates a positive feedback loop in which credible clusters expand, attract ties, and further improve the local payoff environment for credible participation.

Under the attention economy, the same structural mechanism works in the opposite direction. Opportunistic nodes obtain high returns, spread through imitation, and eventually occupy nearly every active path. Although non-opportunistic nodes are more dissatisfied with opportunistic ties, the payoff advantage is too strong for the modest rewiring rate to reverse the regime. Under blanket regulation, nodes become silent before a credible active backbone can form. Rewiring cannot restore communication because tie adaptation changes partners, not the low payoff of active participation. This comparison shows that network adaptation can reinforce incentive changes but cannot substitute for a favorable payoff structure.

### 3.6 Robustness across network structures, rewiring rates, and population sizes

**Figure 7:**
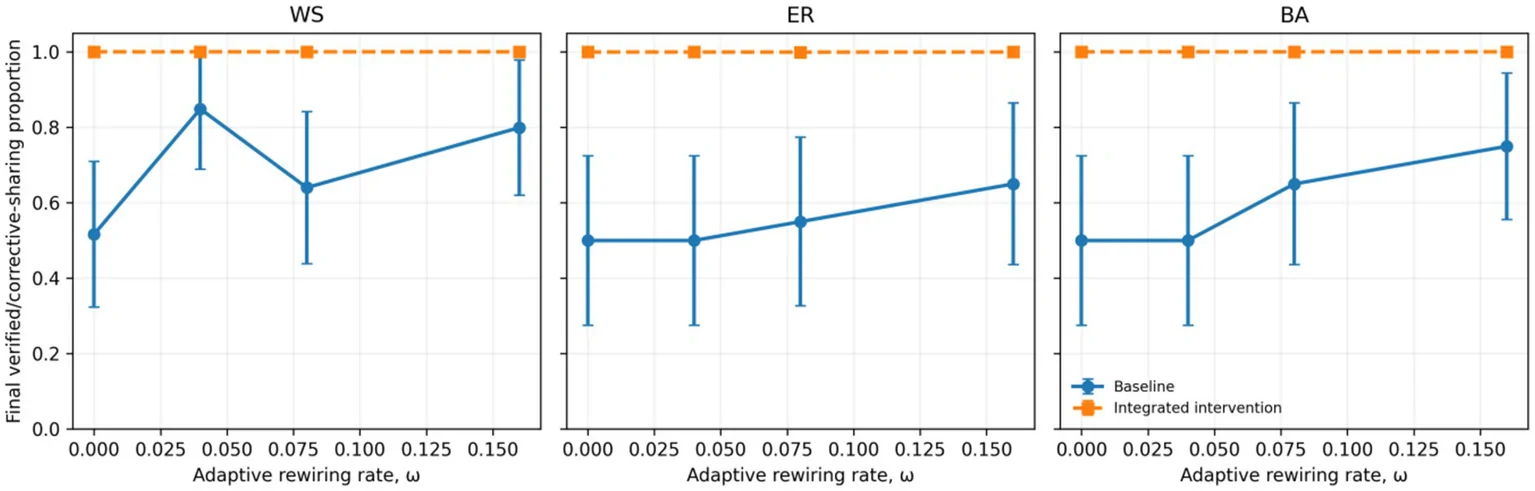
Robustness across network topologies and adaptive rewiring rates. Points show mean final verified/corrective-sharing proportions and error bars show 95% Monte Carlo confidence intervals. The baseline remains sensitive to topology and stochastic local structure because it is intentionally bistable, whereas the integrated intervention remains near complete credible dominance across small-world, random, and scale-free networks and across all tested rewiring rates.

Figure 7 compares the baseline and integrated intervention across small-world, Erdős–Rényi, and Barabási–Albert networks and four rewiring rates. The baseline remains sensitive to topology and stochastic local structure. This is expected from the analytical bistability: changes in clustering, degree heterogeneity, and early local composition alter which active strategy obtains an initial advantage. Rewiring changes the probability of reaching credible dominance but does not eliminate uncertainty across every baseline condition.

The integrated intervention is qualitatively different. Its final credible share is at least 0.9998 across all tested topologies and rewiring rates, including ω=0 , which removes adaptive tie change. This result shows that the intervention is not successful only because the rewiring rule favors credible nodes. The payoff configuration itself makes credible sharing invasion-resistant, while adaptive rewiring accelerates and structurally consolidates the outcome. The topology comparison also indicates that the main conclusion does not depend on the small-world assumption.

Increasing ω has its largest influence when payoffs are competitive. Under extreme attention-economy or silence-inducing conditions, modest structural adaptation cannot overcome the strong behavioral advantage of the dominant strategy. Under the integrated intervention, the endpoint is already robust and additional rewiring mainly affects the transient organization of ties. The results therefore support a conditional interpretation: network governance is most useful when combined with communication and incentive policies, not as a stand-alone substitute.

Finite-size scaling provides a separate check on population size. The policy-induced regimes are invariant when the network is halved or doubled: the attention-economy scenario converges to *O* = 1.000, blanket regulation to *S* = 1.000, and the integrated intervention to *C* = 1.000 at *N* = 125, 250, and 500. Their public-health network outcomes are likewise unchanged: the attention economy remains fully connected but opportunistic, blanket regulation produces a complete information vacuum with no active giant component, and the integrated intervention produces complete verified exposure with full active connectivity. The deliberately near-boundary baseline remains stochastic but credible sharing is dominant on average at every size, increasing from *C* = 0.798 at *N* = 125 to 0.884 at *N* = 250 and 0.943 at *N* = 500; its between-run standard deviation correspondingly declines from 0.410 to 0.311 and 0.224. Across all three sizes, the baseline retains zero information vacuum and a fully connected active component. Thus, population size affects basin occupancy and stochastic dispersion in the bistable baseline but does not change the qualitative identity of any tested regime. Table 8 summarizes the regime-level comparison across the three population sizes.

**Table 8:** Finite-size scaling validation across representative regimes.

Scenario/reported strategy	*N* = 125	*N* = 250	*N* = 500
Baseline—C	0.798	0.884	0.943
Attention economy—O	1.000	1.000	1.000
Blanket regulation—S	1.000	1.000	1.000
Integrated intervention—C	1.000	1.000	1.000

## 4 Discussion

### 4.1 Principal findings

This study develops an interaction-dependent and network-adaptive explanation of public information sharing during health emergencies. Four findings are central.

First, credible information sharing, opportunistic dissemination, and silence are not simply high-, medium-, and low-participation states. They are distinct strategic regimes generated by different combinations of public value, private incentives, participation costs, and neighborhood feedback. Verified/corrective sharing benefits from coordination with similar users; opportunistic dissemination benefits from echo-chamber reinforcement; and silence benefits from avoidance but becomes socially costly when it creates information vacuums. The three-strategy formulation therefore captures a mechanism that binary true–false or share–not-share models overlook: harmful information environments can emerge through excessive activity or through insufficient activity.

Second, the regulation results clarify why targeted accountability and broad communication pressure should not be treated as equivalent. Raising the expected cost of opportunistic dissemination and strengthening corrective pressure shifts the system toward credible sharing. Raising generalized liability for good-faith dissemination weakens the same strategy that public health authorities depend on for community reach. When both active strategies are heavily burdened, the system does not become orderly; it becomes silent. This mechanism is consistent with risk-communication principles that emphasize trust, clarity, and enabling informed action rather than relying only on suppression.

Third, the simulations separate structural activity from information quality. Under attention-economy conditions, the network is fully connected and communication remains intense, but verified-information exposure is zero. A conventional network analysis focused only on density, reach, or giant-component size could therefore misclassify an opportunistic information environment as resilient. Conversely, blanket regulation preserves potential social ties while eliminating active dissemination. Public-health evaluation should accordingly measure what people are exposed to and whether credible information reaches them, not merely whether messages and links exist.

Fourth, the integrated intervention is substantially more robust than any isolated mechanism. Increasing information value, reducing verification and good-faith liability costs, weakening private attention returns, imposing targeted sanctions, and increasing the opportunity cost of silence jointly produce rapid credible-sharing dominance. This result supports a portfolio approach to infodemic management. Message improvement, verification support, platform incentive design, targeted accountability, and community engagement are complements rather than substitutes.

The finite-size scaling results further delimit this conclusion. Clear intervention-induced regimes persist when N is halved or doubled, whereas the intentionally bistable baseline shows size-dependent basin occupancy and declining stochastic dispersion as N increases. This pattern is consistent with the model’s path-dependence mechanism rather than with a population-size-specific intervention effect.

The findings also clarify how the present results relate to earlier work. Evidence on health-information sharing has shown that message usefulness, source cues, media literacy, and accuracy prompts can improve the quality of sharing (10, 12–14), while overload and perceived responsibility can lead to information avoidance (9). The present model does not replace these findings; it connects them by showing how the same mechanisms can compete within one evolving information environment. The attention-economy result is consistent with research on false-information diffusion, echo chambers, and the amplification of emotionally salient content (7, 8, 30, 31), but adds a network-level qualification: high connectivity can coexist with very poor information quality. Likewise, established network research shows that tie strength, clustering, and topology shape diffusion (15–22). Our results extend this line by treating ties as responsive to accumulated credibility rather than as a fixed background condition.

Recent dynamic studies point in the same general direction but operate at different levels of analysis. Lv et al. (26) document stage-dependent evolution in public-health information collaboration networks; Qiu et al. (27) show that social norms and indirect ties can redirect crisis-response behavior; and Xu et al. (28) demonstrate how changing incentives alter strategic choices in a public-health governance setting. The present framework extends these contributions by placing three competing information-sharing strategies and adaptive communication ties in the same model. Its regulatory result also qualifies a simple suppression logic: stronger accountability can improve the information environment when it is directed at opportunistic dissemination, but broader liability can weaken the good-faith participation that corrective communication requires. This distinction is consistent with the emphasis of risk-communication guidance on trusted, actionable, and enabling communication (1–4, 29).

### 4.2 Theoretical contributions

The study contributes to risk communication and infodemic research in four respects. The first contribution is microfoundational. Empirical studies have shown that information usefulness, trusted sources, message features, media literacy, emotional content, and verification prompts influence health-information sharing. The present model organizes these mechanisms within a single payoff structure and explains how they compete over time. Importantly, the model does not presume that unverified sharing is always irrational. Opportunistic dissemination can be individually advantageous when attention rewards and peer amplification exceed expected sanctions and correction costs.

The second contribution concerns endogenous network structure. Existing research demonstrates that tie strength, homophily, centrality, echo chambers, and network clustering shape information diffusion. Adaptive-network theory further shows that node states and relationships can coevolve. By integrating trust-biased rewiring with strategy imitation, the model represents credibility as both a behavioral and structural process. Credible sharing changes who remains connected to whom, and those ties subsequently alter strategy payoffs and exposure. This network-based mechanism also provides a useful point of connection with recent work on group-level reputation and cooperation. Studies of collective reputation show that cooperative behavior may depend not only on the reputation of individual interaction partners but also on evaluations formed at the group level (39). The present model operates primarily through local individual interactions: nodes compare neighboring payoffs, imitate locally successful strategies, and preferentially rewire toward more credible or responsive partners. Nevertheless, repeated verified or opportunistic behavior can generate clusters with systematically different credibility profiles, suggesting an emergent group-level analogue of reputation. Introducing collective reputation explicitly would add a meso-level mechanism between individual behavior and network structure. For example, the perceived reputation of a communication community could influence partner-selection probabilities, the expected credibility of information received from its members, or the effectiveness of corrective behavior. Such an extension would allow future models to examine whether group reputation reinforces credible-information clusters, accelerates segregation between credible and opportunistic communities, or enables cooperation to persist when individual verification is costly.

The third contribution is evaluative. Public health communication aims to support informed decisions and protective behavior, while infodemic management seeks to reduce harmful information and identify unmet information needs. The four network outcomes used here—verified exposure, opportunistic exposure, information vacuum, and active connectivity—more closely reflect these goals than generic diffusion volume. They also reveal two failure modes that require different interventions: an active but opportunistic network and an inactive network characterized by silence.

The fourth contribution concerns regulatory asymmetry. Targeted sanctions and generalized liability are often discussed together as forms of communication control, but they act on different strategies in the model. Separating them shows why suppressing opportunistic dissemination and sustaining credible participation are related but not identical governance tasks. A policy can reduce the relative return to opportunistic sharing without burdening credible users, whereas broad responsibility pressure can weaken the corrective participation on which public-health communication also depends.

### 4.3 Implications for public-health risk communication and infodemic management

The results suggest five practical priorities. First, health authorities should increase V by making messages timely, locally relevant, actionable, and explicit about uncertainty. Research on messaging during public health emergencies indicates that content and deployment strategy influence dissemination, while WHO guidance emphasizes communication that enables informed protective decisions. Messages that clearly explain what is known, what remains uncertain, and what the public can do are more likely to generate public value than generic warnings.

Second, authorities and platforms should reduce c by providing pre-verified, easily shareable materials; visible source and date labels; concise evidence summaries; and prompts that support accuracy checking before forwarding. Experimental evidence indicates that accuracy nudges can stimulate verification and reduce health-misinformation sharing. Community organizations and health professionals can further reduce cognitive burden by translating technical guidance into culturally and locally meaningful formats.

Third, regulation should increase f and g without unnecessarily increasing l . Targeted measures can include transparent labeling, friction for repeatedly debunked claims, proportionate sanctions for demonstrably harmful or deceptive dissemination, and rapid corrective responses from credible actors. At the same time, good-faith users need clear responsibility boundaries and safe channels for sharing official or verified information. Ambiguous rules that make all emergency discussion appear risky may generate a chilling effect and reduce the corrective capacity of the public.

Fourth, platform incentive design must address m and q . High engagement rewards can make sensational or emotionally charged content privately profitable even when users recognize its uncertain quality. Reducing algorithmic amplification of repeatedly unreliable content, limiting monetization incentives during emergencies, and diversifying exposure beyond homogeneous clusters can weaken opportunistic reinforcement. These interventions should be evaluated not only by content removal counts but also by changes in verified and opportunistic exposure.

Fifth, silence requires active prevention. Information avoidance can result from overload, anxiety, low trust, or perceived participation risk. Public-health agencies should therefore monitor information vacuums, invite questions, acknowledge uncertainty, respond to community concerns, and work through trusted local intermediaries. The model indicates that silence is not corrected simply by suppressing misinformation. It is reduced when credible participation is valuable, manageable, and safe.

### 4.4 Limitations and future research

The model is intentionally stylized and has several limitations. First, the parameters are mechanism-oriented and normalized rather than estimated from a specific event. The results establish internally consistent regime relationships but do not provide event-specific causal estimates. Future research should calibrate parameters using survey experiments, digital-trace data, message-level sharing records, or longitudinal public-opinion data from outbreaks, vaccination campaigns, food-safety incidents, or environmental health emergencies.

Second, three strategies cannot capture the full heterogeneity of information behavior. Users may selectively share within strong ties, delay forwarding, express uncertainty, consume without disseminating, correct privately, or switch among multiple content types. Extensions could introduce conditional verification, selective audiences, emotional amplification, automated accounts, and professional or institutional actors.

Third, the network is undirected and single-layered, whereas real communication systems combine follower networks, private groups, face-to-face ties, news media, health institutions, and platform algorithms. Directed, weighted, temporal, and multiplex networks would allow source authority, asymmetric exposure, and cross-platform migration to be represented more realistically. Future extensions could also introduce group-level reputation to examine how collective credibility interacts with individual strategy updating and adaptive network formation.

Fourth, the rewiring rule and the parameters governing trust-biased adaptation are theoretical representations rather than quantities calibrated from observed tie-change data. In particular, the adaptive rewiring rate ω, trust preference η, and dissatisfaction weights used to trigger tie revision are specified to represent plausible relative tendencies within the model and should not be interpreted as empirically estimated probabilities of unfollowing, blocking, tie strengthening, or partner selection. Actual tie adaptation may depend on ideology, identity, platform design, relationship costs, and emergency context. Future research should calibrate these parameters using longitudinal digital-trace data, platform interaction records, or experimental evidence on tie formation and dissolution.

Fifth, the model evaluates the health information environment but does not directly couple information exposure to disease transmission or protective behavior. A future coupled information–behavior–epidemic model could test how verified exposure, opportunistic exposure, and information vacuums affect vaccination, distancing, treatment seeking, or other health outcomes. Cross-national and cross-cultural comparisons would also be valuable because liability perceptions, institutional trust, platform governance, and community networks vary substantially.

Sixth, the framework has a defined scope of application. It is most suitable for public health emergencies in which members of the public participate in decentralized, many-to-many digital or interpersonal communication, can choose whether and how to share information, and can alter their communication ties. It is less directly applicable to highly centralized one-way warning systems, professional command networks in which communication is role-mandated, or settings where connectivity is too limited for local network effects to develop. The interpretation of the parameters may also differ across outbreaks, vaccination controversies, food-safety incidents, and environmental health emergencies because institutional trust, platform design, legal responsibility, and information urgency vary. Extension to non-health emergencies should therefore depend on whether comparable incentive and network mechanisms are present rather than being assumed from the present results.

Seventh, the integrated-intervention scenario changes multiple model parameters at the same time and is designed to represent a complete policy portfolio rather than an attribution design. Its favorable outcome therefore reflects the aggregate effect of the specified package, and the independent marginal contribution of message improvement, verification support, targeted accountability, platform-incentive adjustment, or anti-withdrawal measures cannot be disentangled from this scenario. A future factorial or ablation analysis that varies these components separately and in combination would be required to estimate standalone effects and policy interactions.

## 5 Conclusion

Information sharing during public health emergencies is a strategic and networked process, not a fixed-probability diffusion mechanism. Interaction-dependent payoffs generate three distinct regimes: verified/corrective coordination, opportunistic amplification, and strategic silence. Adaptive communication ties reinforce local credibility patterns, but network connectivity alone does not indicate a healthy information environment. A network can be highly connected and entirely opportunistic, or socially connected yet communicatively silent.

The most consequential policy result is the distinction between targeted accountability and generalized participation pressure. Sanctions and corrective mechanisms directed at opportunistic dissemination can support credible sharing, whereas generalized liability weakens good-faith participation and can contribute to information vacuums when broader withdrawal incentives are also present. The most robust outcome arises from an integrated intervention that increases the value and shareability of credible information, lowers verification and good-faith liability costs, reduces private attention returns, and maintains targeted constraints on opportunistic content. These findings provide a mechanism-based foundation for designing risk communication and infodemic-management strategies that promote credible public participation without inducing withdrawal.

## Data Availability

The original contributions presented in the study are included in the article/Supplementary material, further inquiries can be directed to the corresponding author.
